# Transfer Learning-Based Hyperspectral Image Classification Using Residual Dense Connection Networks

**DOI:** 10.3390/s24092664

**Published:** 2024-04-23

**Authors:** Hao Zhou, Xianwang Wang, Kunming Xia, Yi Ma, Guowu Yuan

**Affiliations:** 1 School of Information Science and Engineering, Yunnan University, Kunming 650504, China; zhouhao@ynu.edu.cn (H.Z.); wangxianwang@mail.ynu.edu.cn (X.W.); xiakunming@stu.ynu.edu.cn (K.X.); 2 Yunnan Power Grid Co., Ltd., Kunming 650011, China; mayi@dlyjy.yn.csg.cn

**Keywords:** hyperspectral image, cross-domain few-shot learning, transfer learning, residual dense connection network, spatial–spectral features

## Abstract

The extraction of effective classification features from high-dimensional hyperspectral images, impeded by the scarcity of labeled samples and uneven sample distribution, represents a formidable challenge within hyperspectral image classification. Traditional few-shot learning methods confront the dual dilemma of limited annotated samples and the necessity for deeper, more effective features from complex hyperspectral data, often resulting in suboptimal outcomes. The prohibitive cost of sample annotation further exacerbates the challenge, making it difficult to rely on a scant number of annotated samples for effective feature extraction. Prevailing high-accuracy algorithms require abundant annotated samples and falter in deriving deep, discriminative features from limited data, compromising classification performance for complex substances. This paper advocates for an integration of advanced spectral–spatial feature extraction with meta-transfer learning to address the classification of hyperspectral signals amidst insufficient labeled samples. Initially trained on a source domain dataset with ample labels, the model undergoes transference to a target domain with minimal samples, utilizing dense connection blocks and tree-dimensional convolutional residual connections to enhance feature extraction and maximize spatial and spectral information retrieval. This approach, validated on three diverse hyperspectral datasets—IP, UP, and Salinas—significantly surpasses existing classification algorithms and small-sample techniques in accuracy, demonstrating its applicability to high-dimensional signal classification under label constraints.

## 1. Introduction

Hyperspectral imaging (HSI) systems amass extensive spatial and spectral data across a broad array of spectral bands, presenting a rich tapestry of information [1,2]. This bounty has catalyzed advancements across varied domains, such as precision agriculture [3], environmental surveillance [4,5], and disaster mitigation [6,7], signifying its interdisciplinary impact. The realm of hyperspectral image classification, a pivotal segment of hyperspectral analysis, has elicited considerable scholarly interest [8,9]. Yet, the classification endeavors for hyperspectral remote-sensing imagery confront persistent obstacles. A critical imperative lies in the more profound exploration of the intrinsic deep features within hyperspectral images. Addressing the paucity of training samples and enhancing classification efficacy in high-dimensional contexts with limited data remain pressing challenges. These hurdles underscore the substantial prospects for continued research and advancements in the field.

In traditional classification methods, the classification of hyperspectral images has focused on manual feature extraction [9,10,11,12] and the use of traditional shallow classifiers, including K-Nearest Neighbor (KNN) [13], Support Vector Machine (SVM) [14], logistic regression [15], the manifold learning method [16], among others. These conventional methods can only extract shallow feature information and neglect deep feature information. Classification performance relies significantly on prior knowledge, manual parameter adjustments, and feature selection. However, this approach lacks the adaptability required to address classification tasks in complex scenarios.

Deep learning methods possess the ability to acquire discriminative features from extensive annotated data and apply these features to classification tasks. As a result, deep learning methods have emerged as a promising approach to hyperspectral image (HSI) classification, offering substantial advantages over traditional methods. Chen et al. [17] utilized deep stacked autoencoders to extract spatial and spectral features from hyperspectral images. This approach effectively captured contextual spatial information and spectral information from HSIs, leading to a successful classification and good performance. To address the distinct characteristics of hyperspectral image data cubes, Li et al. [18] employed a 3D convolutional neural network (3D-CNN) for hyperspectral image classification. Thompson et al. used deep belief networks to extract features at a deep level for hyperspectral image classification [19]. Zhong et al. [20] introduced a supervised spectral–spatial residual network (SSRN) to iteratively acquire discriminative features from the abundant spectral characteristics and spatial contexts of hyperspectral images (HSI). The goal was to extract integrated spatial–spectral information and identify significant spectral–spatial features for classification purposes.

The performance of conventional supervised deep learning methods is based on a significant number of labeled samples for model training. Nevertheless, the exorbitant cost of annotation leads to a severely restricted number of labeled samples for hyperspectral images as a whole. Therefore, using traditional deep learning models for hyperspectral image classification with insufficient training samples can easily lead to overfitting and suboptimal classification performance. To overcome this challenge, researchers have proposed various approaches to tackle the issue of hyperspectral image classification in scenarios with limited sample sizes. Some approaches [21,22] employ data augmentation to generate additional training samples for deep learning models such as CNNs, thus expanding data size and improving the model’s generalizability. Several semi-supervised approaches [23,24] involve the combination of a limited number of labeled samples with unlabeled samples during training. These methods leverage the information from unlabeled samples to obtain feature representations that are more robust and highly generalized. Transfer learning-based approaches [25,26] employ a model that has been pre-trained on a large-scale dataset. The weights of the pre-trained model serve as initialization parameters, which are subsequently fine-tuned on a small sample dataset. By harnessing the feature extraction capabilities of the pre-trained model, this approach effectively enhances the classification performance using small-sample datasets.

Taking into account the challenges in hyperspectral image classification, the limited availability of labeled training samples in hyperspectral images poses a significant constraint on the learning and feature extraction capacity of deep neural network models. Furthermore, the high-dimensional characteristics of hyperspectral images present a challenge to models trained on a small number of annotated samples regarding the extraction of an adequate set of features. As a consequence, the extraction of intrinsic deep-level features from hyperspectral images becomes arduous, leading to a diminished classification accuracy in hyperspectral image classification tasks. Therefore, the construction of deep neural network models for hyperspectral image classification in scenarios with limited training samples poses a significant research challenge. We took into account that ResNet, through residual blocks, enables inter-layer connections that reinforce feature reuse and alleviate the vanishing gradient problem, and in DenseNet structures, each layer is directly connected to all subsequent layers, allowing the extraction of deeper features, further mitigating the vanishing gradient problem and effectively extracting deep features. Therefore, to more effectively extract deep features from the spectral and spatial dimensions of hyperspectral images under conditions of limited samples and to enhance the performance of hyperspectral classification, this paper presents a meta-transfer framework for few-shot hyperspectral image classification based on a three-dimensional Residual Dense Connection Network (ResDenseNet). The primary contributions of this paper are summarized as follows.

(1) The proposition of a meta-transfer few-shot learning classification (MFSC) method aimed at surmounting the hurdle of scarce annotated samples: The method employs a meta-learning training strategy to harmonize data from disparate class samples within a unified feature space, facilitating the prediction of categories for unlabeled samples through similarity between the support set and query set within this feature domain.

(2) The introduction of a novel hyperspectral image classification network, dubbed ResDenseNet, designed to address the underutilization of spectral and spatial information within hyperspectral images: This architecture synergizes the DenseNet (Densely Connected Convolutional Networks) [27,28] and ResNet (Residual Network) [29] frameworks. An enhanced spectral dense block is deployed for the assimilation of spatial–spectral features, complemented by a three-dimensional residual block for the further extraction of spatial and spectral attributes. Classification is achieved through a multilayer perceptron (MLP). The ResDenseNet architecture comprehensively mines deep features within the proximal space of samples, extracting more discriminative attributes to bolster the classification acumen of hyperspectral images.

The remainder of this study is structured as follows: Section 2 provides an overview of the existing cross-domain few-shot hyperspectral classification algorithm for transfer learning. In Section 3, we present the framework of our proposed MFSC approach, which aims to tackle the issue of limited labeled samples in hyperspectral images. Section 4 presents the experimental results of our methods, along with our analysis. Finally, Section 5 concludes our work.

## 2. Related Work

In the context of transfer learning [30,31,32], a model is initially trained on a source dataset, which comprises abundant annotated data from multiple classes known as source classes. Subsequently, the model parameters and features are then adapted to the target dataset with a limited number of labeled samples, where the classes are non-overlapping. This process allows the model to be transferred and adjusted to handle the target dataset, which contains only a small number of labeled samples. Koch et al. [32] proposed an early technique known as Deep Convolutional Siamese Networks. This method performs feature extraction on a pair of samples using the same network and employs the Euclidean distance to measure similarity for classification. However, despite its simplicity and intuitiveness, this approach often fails to achieve satisfactory results in complex scenarios. Based on this, Vinyals et al. [33] introduced Matching Networks, which integrate bidirectional LSTM networks with feature metric learning. By calculating the cosine distance between output features, it captures the similarity between support set and query set images, thereby achieving the classification objective. Nevertheless, this approach encounters difficulties when dealing with intricate and irregular spatial structures. Although this method performs well when the distribution of the source domain data is close to that of the target domain data, existing transfer learning methods struggle to effectively generalize the model from the source domain to the target domain when there is a significant difference in data distributions. Therefore, research is conducted on cross-domain small-sample classification techniques for situations where the source and target domain data distributions differ, aiming to bolster the transfer learning model’s capacity for generalization.

To address the challenges posed by cross-task learning, researchers have proposed a range of meta-learning techniques [34,35,36,37,38,39], which can be classified into two main categories: metric-based and optimization-based approaches. Metric-based methods focus on acquiring a robust feature space by employing the Euclidean distance to gauge the likeness between unlabeled samples and labeled samples of each class. Conversely, optimization-based meta-learning strategies aim to train a universal model capable of swiftly converging to an effective solution for new tasks through a limited number of gradient descent iterations. Nevertheless, when dealing with scant training samples, these methods are susceptible to overfitting, and their weight-update process tends to be relatively sluggish. Consequently, there is a pressing need to enhance and refine these meta-learning techniques to ensure their practicality and efficacy within the realm of few-shot learning.

On the other hand, given the high-dimensional characteristics of hyperspectral images, combining more efficient hyperspectral feature extraction methods with small-sample learning techniques has become a pivotal approach to tackle the challenge of limited annotated samples in hyperspectral data. Liu et al. [39] introduced a Deep Few-shot Learning (DFSL) method that explores the impact of various feature extraction methods on the metric space for classification outcomes. However, this approach still faces limitations when dealing with similarity issues within the metric space. Reference [40] proposes a novel and compact framework based on the Transformer, called the Spectral Coordinate Transformer (SCFormer), which employs two mask patterns (Random Mask and Sequential Mask) in SCFormer-R and SCFormer-S, respectively, aiming to generate more distinguishable spectral features using the existing spectral priors.

To tackle the challenges posed by the characteristics of high-dimensional data features in hyperspectral images and the limited number of labeled training samples, which make it difficult to thoroughly explore the deep-level features of hyperspectral images and subsequently result in suboptimal classification accuracy, this paper proposes a novel approach: the meta-transfer few-shot classification method. Furthermore, to enhance the classification of hyperspectral images, a residual dense connection network is introduced. On the one hand, this method facilitates the transfer of the transferable knowledge acquired from a source domain dataset to the target domain with a limited number of samples. This addresses the issue of restricted training samples that hinder the accuracy of classification in deep learning models. On the other hand, by taking advantage of the capabilities of the residual dense connection network, features are used more effectively, and the exchange of features between convolutional layers is intensified, ultimately contributing to an overall improvement in classification accuracy.

## 3. Proposed Meta-Transfer Hyperspectral Image Few-Shot Classification

### 3.1. Proposed MFSC Framework

**Figure 1:**
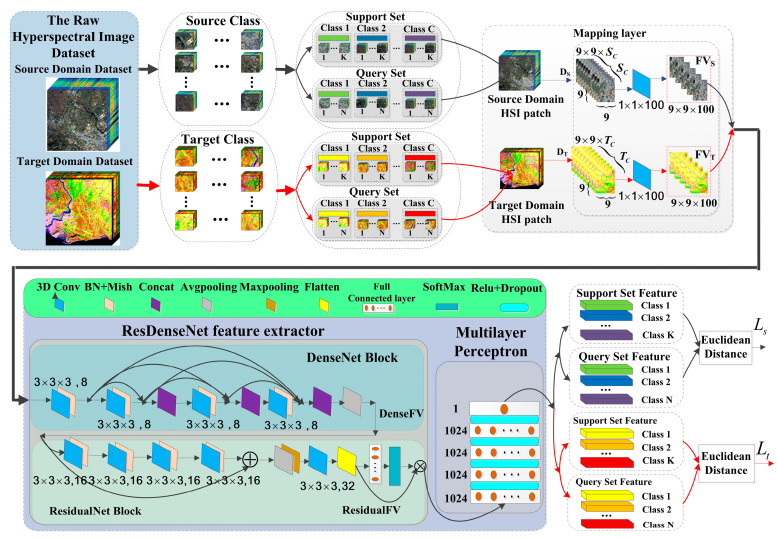
Framework of the proposed MFSC.

The entire process flow diagram is shown in Figure 1. It comprises two main components: the cross-domain few-shot learning strategy and the residual dense connection feature extraction and classification network. Arrows indicate the flow of feature vectors in the algorithm, with red arrows representing feature vectors originating from the target domain, while black arrows represent feature vectors coming from the source domain.

The few-shot learning strategy, based on metric learning-based meta-transfer, leverages the transferrable feature knowledge trained from the source domain dataset and transfers this knowledge to the target domain with a small number of labeled samples. These two types of small-sample learning are conducted simultaneously. Model weights trained on the source domain dataset are used to initialize the weights of the feature extraction network. This is performed to enhance the hyperspectral image (HSI) classification accuracy, addressing the issue of limited training samples that constrain the classification accuracy in deep learning models.

By utilizing the mapping layer and the residual dense connection network, features from the source domain and the target domain are mapped to a feature space. This ensures that samples from the same class have a similar distribution in the feature space, while samples from different classes are distributed as far apart as possible in the feature space. The residual dense connection network allows for the more comprehensive extraction of spatial–spectral features and enhances direct feature transfer between convolutional layers, thus improving classification accuracy.

### 3.2. Cross-Domain Few-Shot Learning and Training Strategy

**Figure 2:**
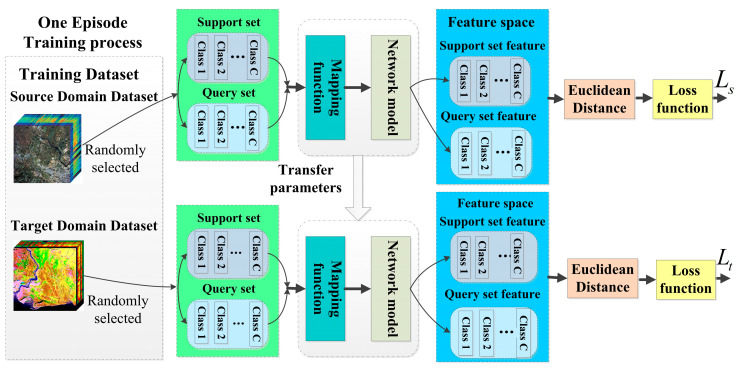
Flowchart of the cross-domain few-shot learning algorithm.

The entire process flowchart for the few-shot learning is shown in Figure 2. Training of the few-shot learning model consists of two stages. First, a set of data called source class data are used to train the model, with this class having an abundant number of samples. Then, training and testing are carried out on the target class data, where the classes do not overlap and only a small number of labeled samples are available. These two stages alternate until the model converges.

From the original HSI datasets of the source and target classes, C classes are randomly selected from each, with each class containing K labeled samples to create the source domain support set $S_{s}={\left\{(xs_{i},ys_{i})\right\}}_{i=1}^{C\times K}$ and the target domain support set $S_{t}={\left\{(xt_{i},yt_{i})\right\}}_{i=1}^{C\times K}$. Then, N unlabeled samples are randomly selected from the remaining data in both the source and target domains to create the source domain query set, $Q_{s}={\left\{\left(xs_{j},ys_{j}\right)\right\}}_{j=1}^{C\times N}$, and the target domain query set, $Q_{t}={\left\{\left(xt_{j},yt_{j}\right)\right\}}_{j=1}^{C\times N}$. This entire selection process is referred to as a C-way K-shot task. Each time the support and query sets are selected for model training, it constitutes an episode.

In each training episode, during the training cycle, the model is first trained on the source domain dataset. The source domain support set, $S_{s}={\left\{(xs_{i},ys_{i})\right\}}_{i=1}^{C\times K}$, is fed into the network to extract features, and the feature vectors, ${cs}_{k}$, for the *k*-th class in the support set in the feature space are computed. The source domain query set samples, $xs_{j}$, are then passed through the feature network to extract embedded features, $f\varphi (xs_{j})$. The Euclidean distance, $d\left(f\varphi \left(xs_{j}\right),{cs}_{k}\right)$, between the feature vectors of the query set samples, $xs_{j}$, and the feature vector, ${cs}_{k}$, of the class to which the support set samples belong in the feature space is calculated [41]. Subsequently, the probability that a query set sample, $xs_{j}$, belongs to class k in the support set is computed using the following the SoftMax function:

(1) $$P\left(y_{j}=k|xs_{j}\in Q_{S}\right)=\frac{\exp\left(-d\left(f\varphi \left(xs_{j}\right),cs_{k}\right)\right)}{\sum_{k=1}^{C}\exp\left(-d\left(f\varphi \left(xs_{j}\right),cs_{k}\right)\right)}$$

In each episode, during the training process, fφ represents a mapping layer and a spatial–spectral feature extraction network with learnable parameters denoted as φ, $y_{j}$ represents the true class labels of the samples ${xs}_{j}$, and C is the number of classes in each episode. The training loss in each episode is calculated as the sum of the negative log probabilities between all query set samples and their corresponding true class labels:

(2) $$L_{s}=\left[-\sum_{\left(xs_{j},ys_{j}\right)\in Q_{s}}\log p_{\varphi }\left(ys_{j}=k|xs_{j}\right)\right]$$

Then, the model continues training using the target domain data. The support set data, $S_{t}={\left\{(xt_{i},yt_{i})\right\}}_{i=1}^{C\times K}$, from the target domain dataset are fed into the model trained on the source domain data. This calculates the feature vector, *ct_k_*, for the *k*-th class in the feature space. Similarly, the samples *xt_j_* from the target domain query set, $Q_{t}={\left\{\left({xt}_{j},{yt}_{j}\right)\right\}}_{j=1}^{C\times N}$, are input into the feature extraction network, extracting embedded features, $f\varphi (xt_{j})$, for the query set samples. The Euclidean distance, $d\left(f\varphi \left({xt}_{j}\right),{ct}_{k}\right)$, between the query sample *xt_j_* and the feature vectors of the samples belonging to class *k* in the feature space is computed. The probability that the query sample *xt_j_* belongs to class *k* is calculated through the SoftMax function. On this basis, the loss value for the query sample is also computed.

(3) $$L_{t}=\left[-\sum_{\left(xt_{j},yt_{j}\right)\in Qt}\log p_{\varphi }\left(yt_{j}=k|xt_{j}\right)\right]$$

The data from the source domain and the target domain are randomly selected to form a training dataset that includes support and query sets. The model is trained by minimizing the loss function and optimizing the parameters of the model. This ensures that the features $f\varphi (xs_{j})$ and $f\varphi (xt_{i})$ of the query samples from the source domain and target domain, respectively, are as close as possible to the corresponding support set features, $cs_{k}$ and $ct_{k}$, for that sample. The minimization of the loss function, J(φ), is calculated using Equation (4).

(4) $$J(\varphi )=-logP(y_{j}=k|x_{j}\in Q_{s})=d(f\varphi (x_{j}),c_{k})+log\sum_{k=1}^{C}e^{\left(-d(f\varphi (x_{j}),c_{k})\right)}$$

After multiple rounds of training with multiple episodes and models, when the loss function in the target domain meets the termination condition, the training is concluded.

### 3.3. Spatial–Spectral Feature Extraction Module Based on ResDenseNet Network

The proposed algorithm workflow is illustrated in Figure 1, which shows the MFSC framework. It mainly consists of three parts: the mapping layer module, the ResDenseNet feature extractor, and the multilayer perceptron module.

#### 3.3.1. Mapping Layer Module

In the mapping module, first 9×9×Sc data cubes, $D_{S}$, are selected from the source dataset as the network’s input, where 9×9 represents the spatial dimensions, and Sc represents the number of spectral bands. For the target domain dataset, $9\times 9\times T_{C}$ data cubes, $D_{T}$, are selected as input for network testing, where $T_{C}$ represents the number of spectral bands. Mapping layers are used to reduce the dimensionality of the input samples, ensuring that the input dimensions are the same. Due to the large number of spectral bands in HSI and strong correlations between adjacent bands, mapping layers use a 1×1×100 convolutional kernel to reduce the number of spectral bands in both the source and target domains, reducing the data to 100 dimensions for convenience in subsequent convolution calculations. The final output of the mapping layer is a support feature vector or a query set feature vector with a size of 9×9×100.

#### 3.3.2. ResDenseNet Feature Extractor

The ResDenseNet feature extractor is used as the spatial–spectral feature extraction network; it consists mainly of a DenseNet module and ResidualNet module. In order to address the loss of feature information due to gradient vanishing, amplify feature propagation, and extract feature vectors more effectively, the algorithm initially employs DenseNet module for model training.

The spectral dense block consists of four sets of convolutional kernels, with each set containing 8 filters of size 3×3×3. These are combined with Mish activation functions and batch normalization (BN) to perform non-linear transformations on the feature maps. In DenseNet, each layer is concatenated with all preceding layers along the channel dimension, combining feature maps from all previous layers as input for the next layer to achieve feature reuse and enhance efficiency:

(5) $$DX_{l}=DH\left(\left[DX_{0},DX_{1},\cdots DX_{l-1}\right]\right)$$

where $DH\left(•\right)$ is a non-linear transformation function, which uses the structure of Convolution 3×3×3 (Conv), batch normalization (BN), Mish, and concatenation operations. The subscript l denotes the layer number. The ReLU function causes some neurons to have an output of 0, resulting in network sparsity, and the Mish [42] function, $f\left(x\right)=xtanh\left(ln\left(1+ex\right)\right)$, unlike the ReLU function, has a softer zero boundary and smoother characteristics, allowing for a better flow of information into deep neural networks and better preservation of information, thus producing enhanced accuracy and generalization. The output of the function is not affected by saturation, and positive values can reach arbitrarily high values, avoiding saturation due to a cap. Therefore, Mish is used as the activation function in this paper. The output feature map from the last layer of dense connection block undergoes average pooling, yielding a vector, DenseFV, of dimensions 8×7×7×100. Subsequently, this vector is fed into the three-dimensional ResidualNet module.

In the ResidualNet module, there are four sets of non-linear transformation functions. Each set of non-linear transformation functions includes 16 filters of size 3 × 3 × 3, batch normalization (BN), and Mish activation. It employs a shortcut connection structure, creating a skip connection between the input of the first layer and the output of the last layer. This design allows the network to concentrate on learning the disparity between input and output, streamlining the learning objectives and challenges. The output feature map of the residual block is of size 16 × 7 × 7 × 100. After undergoing average pooling, max pooling, and a set of 32 filters of size 3×3×3, the feature map is flattened to a 1×1×160 vector (ResidualFV). This vector is then processed through a fully connected layer and a SoftMax activation function. Additionally, it undergoes a multilinear mapping as input to the MLP. The number of nodes in the fully connected layer corresponds to the number of classes in the dataset.

#### 3.3.3. Multilayer Perceptron Module

The ultimately extracted feature vector from the multilinear mapping is fed into the MLP for classification. This MLP consists of five fully connected layers, with the first four layers each containing 1024 nodes. The final fully connected layer has only one node. ReLU activation functions and dropout are incorporated between adjacent fully connected layers. The ultimate output of the multilayer perceptron is employed to compute the loss value following Formula (4), after which the classification process is executed.

Through training, the loss function of the spatial–spectral feature extraction network model is minimized. This optimization of parameters in the residual dense connection module allows it to extract features from the input sample data, mapping them into feature space. In this space, the feature vectors of samples with the same class are closer to each other, resulting in smaller interclass distances, while the feature vectors of samples from different classes are farther apart, leading to larger interclass distances.

## 4. Experiments

### 4.1. Experimental Dataset

**Figure 3:**
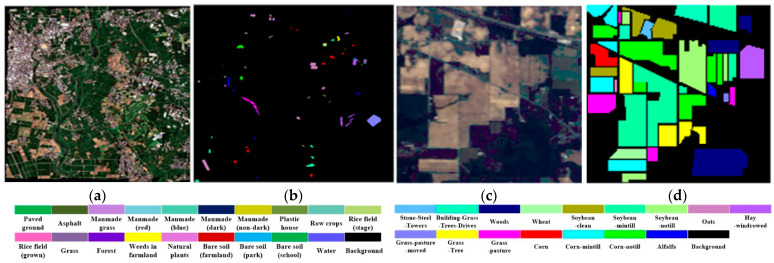
Chikusei and Indian Pines dataset. (**a**) False color image of the Chikusei dataset. (**b**) Ground-truth map of the Chikusei dataset. (**c**) False color image of Indian Pines dataset. (**d**) Ground-truth map of Indian Pines dataset.

**Figure 4:**
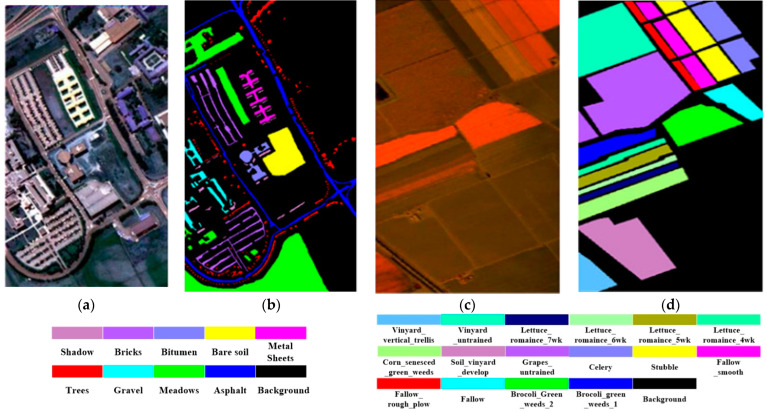
Pavia University and Salinas dataset. (**a**) False color image of the Pavia University dataset. (**b**) Ground-truth map of the Pavia University dataset. (**c**) False color image of the Salinas dataset. (**d**) Ground truth map of the Salinas dataset.

To validate the effectiveness of our approach, we utilized the hyperspectral Chikusei dataset as the source domain dataset, and the Indian Pines, Pavia University, and Salinas datasets [43,44] as the target domain datasets. The pseudo-color images and real land cover maps of this experimental dataset are shown in Figure 3 and Figure 4.

The Chikusei dataset has a spectral wavelength range of 343–1080 nm, a spatial resolution of approximately 2.5 m, and a data size of 2571×2335 pixels. It consists of 128 spectral bands and includes 77,592 ground pixels, categorized into 19 distinct land cover classes.

The Indian Pines dataset covers a spectral wavelength range of 400–2500 nm, with a spatial resolution of about 20 m. The image data size is 145 × 145 pixels and comprises 200 spectral bands. It encompasses a total of 16 land cover classes. The Salinas dataset has a spectral wavelength range of 400–2500 nm and a spatial resolution of approximately 3.7 m. The image size for this dataset is 512 × 217 pixels and includes 224 spectral bands. However, due to the impact of water vapor absorption on certain bands, only 204 bands are retained. This dataset covers 16 different categories of agricultural land cover, including, but not limited to, corn, wheat, soybeans, grasslands, and vineyards. The Pavia University dataset’s spectral wavelength range is 430–860 nm, with a spatial resolution of approximately 1.3 m. After preprocessing, the dataset has a total of 115 spectral bands, with 13 noisy bands removed. Land cover types in this region consist of nine classes, including asphalt roads, meadows, gravel, trees, metals, bare land, asphalt roofs, bricks, and shadows.

### 4.2. Experimental Settings

To evaluate the effectiveness of the MFSC method, 9×9×C data cubes were selected as input for the network from the Chikusei source domain dataset, where 9×9 represents the spatial dimensions, and C is the number of spectral bands. For the target domain datasets, namely Indian Pines, Pavia University, and Salinas, 9×9×L cubes were chosen as the input for testing, where L is the number of spectral bands. The model was trained for 10,000 episodes, and for each episode iteration, following the few-shot training method, 1 labeled sample and 19 unlabeled samples from each class were randomly selected to form the source dataset for model training. The Adam optimizer was used, and to balance convergence speed and accuracy, the model learning rate was set to 0.001. Furthermore, to account for the impact of random sample selection on model training, all experimental results were averaged over 10 trials. The hardware environment used for this experiment is a laptop equipped with an Intel Core i7-4810MQ 8-core 2.80 GHz processor, 16 GB of memory, and an NVIDIA GeForce RTX 2060 graphics card with 6 GB RAM, while the software environment utilized Python 3.8 and PyTorch 1.7.1 running on Windows 10.

### 4.3. Experimental Results and Analysis

To validate the effectiveness of the proposed method in the paper, it was compared with non-few-shot learning methods and few-shot learning methods. In experiments comparing the proposed method with non-few-shot learning methods, the proposed method was compared with SVM, 3D-CNN [45], and SSRN [46]. In experiments comparing the proposed method with other few-shot learning methods, the proposed method was compared with the DFSL + NN [37], DFSL + SVM [47,48], RN-FSL [49], Gai-CFSL [50], DPGN [51], DCFSL [52], SCFormer-R, and SCFormer-S [41] methods. In each comparison experiment, the same training approach as the few-shot methods was employed. Five labeled samples from each class in the target domain dataset were randomly selected for transferring the model trained in the source domain to the target domain, with the remaining target domain samples used as test data. For the small-sample learning methods in comparison, we randomly selected 200 labeled source domain samples from each class to learn transferable knowledge, following the same setup for comparison. To verify the effectiveness of the Mish function and batch normalization (BN) added to the model in the paper, a comparative performance analysis was performed using the DCFSL method. In this comparison, the Mish + BN part was removed, while keeping the rest of the network structure consistent, serving as a set of ablation experiments. The results of the ablation experiments are presented in the “MFSC” row of the tables, where the activation function used is the Softmax activation function, consistent with the DCFSL method. In contrast, the experimental data in the “Ours” row were obtained under the MFSC algorithm framework, incorporating Mish + BN and replacing the original Softmax activation function. For the IP, UP, and Salinas datasets, the study compared the classification performance of different methods. The evaluation was carried out using three metrics: overall accuracy (OA), average accuracy (AA), and Kappa coefficient. Specific comparative results are shown in Table 1, Table 2 and Table 3.

Table 1, Table 2 and Table 3 present the results of comparative experiments on the target datasets, IP, UP, and Salinas, with each class having five labeled samples. From the tables, it can be observed that the methods based on few-shot learning achieve higher overall accuracy compared to non-few-shot methods. This indicates that the episodic training strategy is better suited for classification tasks with limited labeled samples. In the IP dataset, the proposed few-shot learning method shows significant improvements over the traditional SVM classification method, with an increase of 25.64% in OA, 21.95% in AA, and a 28.13% increase in Kappa. In the IP, UP, and Salinas datasets, when compared to deep learning-based methods like 3D-CNN and SSRN, the proposed method achieves significant increases in OA when the number of labeled samples is five, with improvements of 16.73%, 19.35% and 6.34% in IP; and 10.13%, 8.83%, and 4.15% in UP and Salinas, respectively. This indicates that the meta-learning training strategy allows the model to learn transferable knowledge and features from the source-class data, thus aiding in predicting the target-class data. The relatively low performance of the non-few-shot learning methods shown in Table 1, Table 2 and Table 3 illustrates that non-small-sample learning methods extract shallow features with weaker discriminative capabilities for different target categories. The limited labeled samples are insufficient for non-small-sample learning methods to effectively train a classification model. However, meta-learning training strategies enable the model to learn transferable knowledge and features from the source-class data, aiding in predicting target-class data.

In the few-shot classification methods, the method proposed in this paper also demonstrates significant improvements in detection accuracy compared to other methods. On the IP, UP, and Salinas datasets, when compared to the DFSL + NN, DFSL + SVM, RN-FSL, Gai-CFSL, DCFSL, SCFormer-R, and SCFormer-S methods, the proposed method achieves improvements in OA of 12.95%, 10.91%, 14.43%, 8.83%, 5.79%, 7.59%, and 7.65% on IP; 8.27%, 6.39%, 5.84%, 2.9%, 2.37%, 3.71%, and 2.19% on UP; and 3.92%, 4.02%, 6.86%, 3.14%, 1.63% 1.67%, and 2.15% on Salinas, respectively, when there are few labeled samples in the target domain. With the presence of a small number of labeled samples in the target domain, the method proposed in this article utilizes the ResDenseNet network to reduce data distribution differences and learn more discriminative feature spaces. Compared to other methods, this approach can obtain a better feature space, which can improve the classification performance of the target domain samples. The classification results on the IP, UP, and Salinas datasets show that the proposed method achieves average accuracy (OA) of 72.60%, 86.02%, and 90.97%, respectively. This strongly confirms the effectiveness and robustness of the ResDenseNet model in the few-shot high-dimensional spectral data classification task. Additionally, the incorporation of the Mish function and batch normalization (BN) not only effectively mitigates the vanishing gradient problem but also enhances the model’s generalization capabilities. Furthermore, compared to the ReLU function, the Mish function is smoother, leading to an improvement in training stability and average accuracy.

Table 4, Table 5 and Table 6 report the detailed classification results of different classification algorithms on the UP, IP, and Salinas datasets, respectively. The last columns of the tables present the classification accuracy and standard deviation for each class in the dataset based on multiple experiments. It can be observed from Table 4 that, compared to other algorithms, the proposed method achieved the highest recognition rates in three of nine categories. It also performed well in accurately classifying the “Bricks”, “Bitumen”, “Metal sheets”, and “Trees” categories, which were challenging for other methods. The proposed method shows a certain gap from the optimal results among the three categories, including “Gravel”, “Meadows”, and “Asphalt” in the UP dataset, when compared to the methods of contrast. The UP dataset has the highest spatial resolution among the three datasets, but it has the lowest spectral resolution. The data for the three categories are the most prone to generating spectrally similar but different substances. The data in Table 5 and Table 6 illustrate that, compared to other algorithms, the method proposed in the paper achieved the highest recognition rates in 11 out of 16 categories and 10 out of 16 categories, respectively. It significantly improved the classification accuracy for categories like “Grapes_untrained”, “Vinyard_untrained”, and “Soil_vinyard_develop” in the Salines dataset, where other methods had relatively lower accuracy. Furthermore, compared to other methods, the proposed method also substantially increased the classification accuracy of categories like “Grass-pasture”, “Corn”, “Corn-mintill”, “Corn-notill”, and "Woods” in the IP dataset.

**Figure 5:**
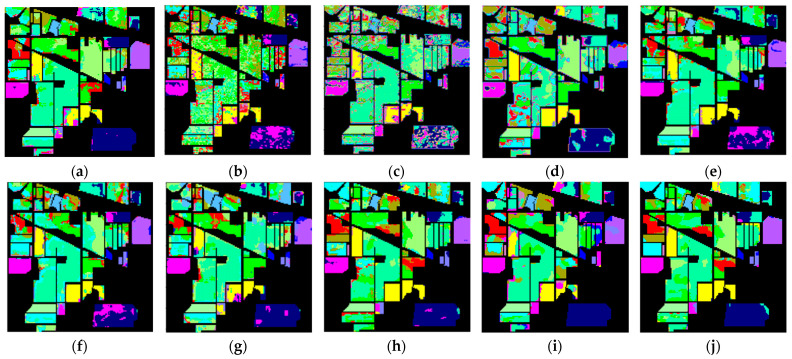
Classification results of different methods in Indian Pines dataset: (**a**) Gai-CFSL; (**b**) SVM; (**c**) 3D-CNN; (**d**) SSRN; (**e**) DFSL + NN; (**f**) DFSL + SVM; (**g**) RN-FSC; (**h**) DCFSL; (**i**) MFSC; and (**j**) MFSC, (Mish + BN) ours.

**Figure 6:**
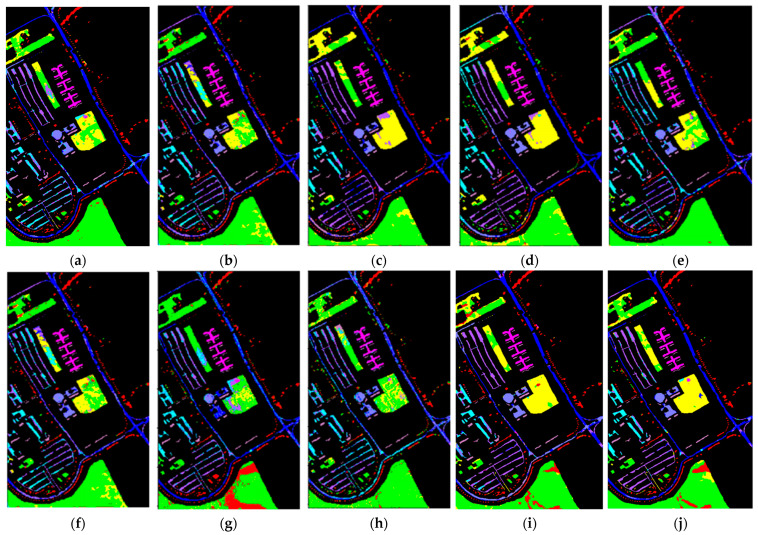
Classification results of different methods in the Pavia University dataset: (**a**) Gai-CFSL; (**b**) SVM; (**c**) 3D-CNN; (**d**) SSRN; (**e**) DFSL + NN; (**f**) DFSL + SVM; (**g**) RN-FSC; (**h**) DCFSL; (**i**) MFSC; and (**j**) MFSC (Mish + BN), ours.

**Figure 7:**
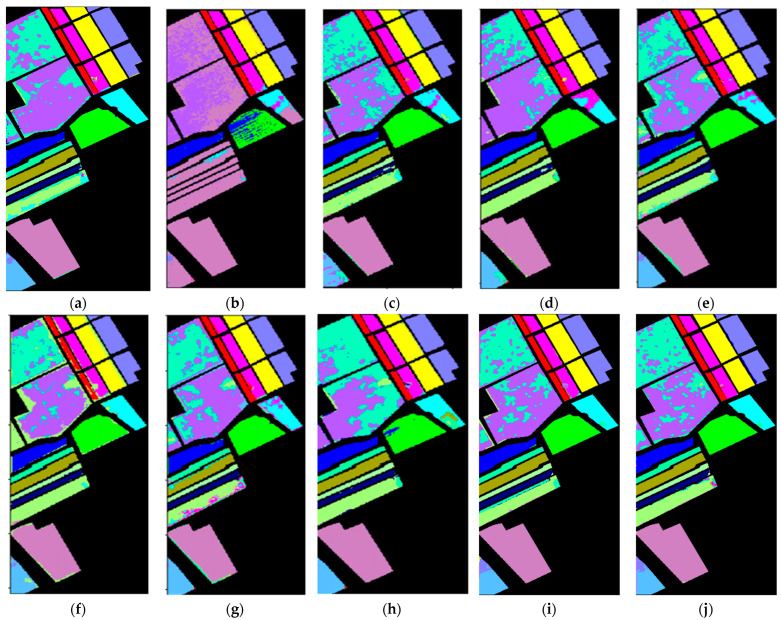
Classification results of different methods in the Salinas dataset: (**a**) Gai-CFSL; (**b**) SVM; (**c**) 3D-CNN; (**d**) SSRN; (**e**) DFSL + NN; (**f**) DFSL + SVM; (**g**) RN-FSC; (**h**) DCFSL; (**i**) MFSC; and (**j**) MFSC (Mish + BN), ours.

Figure 5, Figure 6 and Figure 7 display the classification results of the proposed method and comparative methods using the IP, UP, and Salinas datasets. It can be seen from the figures that the method proposed in this paper exhibits fewer misclassifications. On the contrary, the SVM-based method shows more misclassified objects. Compared to the SVM-based method, the 3D-CNN and SSRN methods have fewer misclassifications, mainly due to the stronger representation learning capabilities of deep learning methods. However, deep learning methods require a large number of training samples, and when the number of training samples is reduced, these methods experience a significant decrease in classification accuracy. This indicates that, when labeled samples are limited, the extracted features are not effective enough, leading to lower accuracy when classifying objects with similar spectral characteristics. In the case of few-shot data, using a few-shot learning approach to construct ResDenseNet significantly improves the classification accuracy compared to the SVM method and deep learning methods like 3D-CNN and SSRN.

In complex scenes, objects within a specific area are rarely composed of just one type of material. Typically, there are varying amounts of other material categories present, leading to spectral noise from other categories within the spectral characteristics of the primary material. Additionally, at the boundaries between two different land cover types, there inevitably exists interference from neighboring land cover categories’ spectral feature vectors. This makes it difficult to accurately extract both the spatial and spectral information of land cover, resulting in subtle differences between different types of land cover. In addition, it can lead to significant distinctions between the same types of land cover, causing the misclassification of certain land cover areas at the boundaries. In the case of few-shot data, while methods like DFSL + NN, DFSL + SVM, and RN-FSC consider the scarcity of labeled samples in hyperspectral imagery, their performance in accurately classifying challenging classes still lags behind the method proposed in this paper.

From the experimental results shown in the figures, it can be observed that when land cover features are relatively easy to distinguish and the feature vectors are distinct, the classification method employed in this paper, as well as other few-shot learning methods, can achieve good classification results. For example, in Figure 5, for the IP dataset, classes like “Oats” and “Grass-Trees”; in Figure 6, for the UP dataset, classes like “Asphalt” and “Shadow”; and in Figure 7, for the Salinas dataset, classes like “Celery”, “Stubble”, “Fallow_smooth”, “Lettuce_romaince_5wk”, and “Brocoil_green_weeds_1” have feature vectors in the feature space that are relatively easy to differentiate. In situations with only a small number of labeled samples, traditional machine learning methods, such as SVM, and general few-shot learning methods can also achieve good classification results. On the contrary, deep learning methods that require a large number of training samples are prone to overfitting, leading to a lower classification accuracy.

For land cover categories with similar features and small feature vector distances that tend to produce errors in classification, such as “Meadows” and “Alfalfa” in the UP dataset; “Vinyard_untrained”, “Vinyard_vertical_trellis”, and “Corn_senesced_green_weeds” in the Salinas dataset; and “Stone-Steel-Tower”, “Hay-windrowed”, “Woods”, and “Soybean-mintill” in the IP dataset, the classification results rely more on the effective extraction of land cover features. From the classification results, it can be seen that the method proposed in this paper achieves a relatively good classification accuracy for such categories. MFSC follows, and DCFSL has fewer misclassifications compared to SVM, 3D-CNN, and SSRN. This indicates, on the one hand, that meta-learning training strategies are advantageous for enhanced knowledge transfer and improved classification performance. On the other hand, it also demonstrates that the residual dense connection network designed in this paper can reduce data distribution differences, leading to a better feature space with higher interclass discriminability. Under small-sample training conditions, the training data’s effectiveness and robustness are superior to those of other methods. Furthermore, the method proposed in this paper has fewer misclassification points than DCFSL, indicating that this network model has good generalizability, can extract deeper and more discriminative features, and can achieve better classification results for classes that are difficult to accurately classify.

## 5. Conclusions

To address the contradiction between the limited number of training samples in HSI (hyperspectral imaging) and the need for a large number of annotated samples for effective deep learning, as well as the trade-off between a small number of labeled samples and the extraction of more effective feature vectors, this paper proposes a hyperspectral image classification method based on the residual dense connection network in the metric learning framework. The main contributions are as follows:

Improved ResDenseNet Network: In comparison to traditional residual networks, this paper introduces a dense connection structure in the three-dimensional convolutional block of the improved ResDenseNet network. This structure can fully explore deep features in the spatial neighborhood of samples, effectively extract spatial and spectral features, and complement the original spectral features. It can obtain more representative features, contributing to hyperspectral images classification.

Activation function and batch normalization: Building on the original network, the ReLU activation function is replaced with the Mish function, and batch normalization (BN) is introduced. This not only effectively alleviates the problem of gradient vanishing; it also enhances the model’s generalization ability. Additionally, compared to the ReLU function, the Mish function is smoother, leading to improvement in training stability and average accuracy.

The experimental results demonstrate that the proposed method, when compared to classical hyperspectral image classification methods and other classic few-shot learning methods, exhibits strong generalization capabilities in deep network models on three datasets: IP, UP, and Salinas. When only a limited number of labeled samples are available, the proposed method achieves a higher recognition accuracy than the algorithms used in the control experiments. Our future work will focus on accurately identifying ground objects in the presence of mixed substances, investigating Transformer learning strategies that can more effectively mine the spatial–spectral features of hyperspectral images, thereby enhancing the classification accuracy of complex ground objects.

## Figures and Tables

**Table 1:** Comparison of the classification performance of different methods in Indian Pines datasets at number of labeled samples K = 5.

	Methods	OA (%)	AA (%)	Kappa × 100
Non-few-shot learning	SVM	45.85	59.24	39.68
	3D-CNN	54.76	63.93	48.72
	SSRN	61.36	59.75	56.91
Few-shot learning	DFSL + NN	59.65	72.24	54.55
	DFSL + SVM	61.69	73.05	56.78
	RN-FSC	58.17	69.90	52.52
	Gai-CFSL	63.77	74.98	59.20
	DCFSL	66.81	77.89	62.64
	SCFormer-R	65.01	74.65	60.20
	SCFormer-S	64.95	75.59	60.31
	MFSC	71.49	81.19	67.81
	MFSC (Mish + BN) Ours	72.60	81.62	69.16

**Table 2:** Comparison of the classification performance of different methods in Pavia University datasets at number of labeled samples K = 5.

	Methods	OA (%)	AA (%)	Kappa × 100
Non-few-shot learning	SVM	64.12	68.18	55.59
	3D-CNN	65.74	73.72	57.37
	SSRN	76.26	79.51	70.56
Few-shot learning	DFSL + NN	77.75	72.24	54.55
	DFSL + SVM	79.63	76.41	73.05
	RN-FSC	80.18	77.12	73.73
	Gai-CFSL	83.12	82.35	77.96
	DCFSL	83.65	83.77	78.70
	SCFormer-R	82.31	82.25	76.55
	SCFormer-S	83.83	82.47	78.47
	MFSC	85.09	86.69	80.78
	MFSC (Mish + BN) Ours	86.02	88.21	81.93

**Table 3:** Comparison of the classification performance of different methods in Salinas datasets at number of labeled samples K = 5.

	Methods	OA (%)	AA (%)	Kappa × 100
Non-few-shot learning	SVM	80.71	87.58	78.61
	3D-CNN	84.20	89.56	82.46
	SSRN	86.39	93.24	84.95
Few-shot learning	DFSL + NN	87.05	91.01	85.63
	DFSL + SVM	86.95	90.08	85.51
	RN-FSC	84.11	88.83	82.38
	Gai-CFSL	87.83	92.41	86.48
	DCFSL	89.34	94.04	88.17
	SCFormer-R	89.30	93.89	88.10
	SCFormer-S	88.82	94.13	87.57
	MFSC	90.54	94.49	89.47
	MFSC (Mish + BN) Ours	90.97	95.36	89.98

**Table 4:** Class-specific classification accuracy (%) of different methods for the target-scene UP datasets (five labeled samples from TD).

Class	SVM	3DCNN	SSRN	DFSL + NN	Gai-CFSL	RN-FSC	DCFSL	SCFormer-R	SCFormer-S	Ours
Shadow	99.13	35.57	98.08	96.92	79.62	99.19	98.66	99.08	95.46	98.52 ± 0.32
Bricks	68.17	57.27	85.34	58.13	88.59	63.48	66.73	74.21	78.15	89.22 ± 0.64
Bitumen	40.62	87.64	60.07	70.62	62.06	70.04	81.18	86.97	81.55	87.28 ± 1.47
Bare soil	37.12	63.40	53.56	71.23	90.66	57.99	77.32	57.50	62.96	87.14 ± 2.45
Metal Sheet	95.44	90.77	98.34	100	98.54	99.43	99.49	98.90	99.01	100 ± 0.0
Trees	60.22	77.31	78.02	89.99	74.65	92.15	93.45	86.54	80.92	94.53 ± 1.59
Gravel	39.98	68.91	55.23	57.47	77.74	49.81	67.46	69.26	71.32	66.19 ± 0.24
Meadows	83.91	63.05	95.13	84.63	71.49	93.44	87.74	90.92	92.04	84.54 ± 1.13
Asphalt	88.98	59.82	91.84	69.19	97.77	68.55	82.20	76.92	80.78	89.42 ± 2.78

**Table 5:** Class-specific classification accuracy (%) of different methods for the Salinas target scene datasets (five labeled samples from TD).

Class	SVM	Res-3D-CNN	SS-CNN	Gai-CFSL	DPGN	RN-FSC	DCFSL	SCFormer-R	SCFormer-S	Ours
Brocoli_green_weeds_1	85.60	39.47	93.02	99.59	87.72	96.45	99.55	98.96	98.22	99.91 ± 0.20
Brocoli_green_weeds_2	98.54	74.02	93.51	98.81	99.49	99.15	99.71	99.87	99.95	99.92 ± 0.14
Fallow	65.38	49.33	84.31	90.18	79.76	85.85	93.68	93.54	98.62	98.55 ± 1.23
Fallow_rough_plow	95.82	88.71	86.43	98.23	98.34	98.49	99.45	98.69	96.26	99.89 ± 0.06
Fallow_smooth	95.83	77.50	90.91	86.75	80.13	82.67	90.39	92.86	96.67	93.48 ± 1.39
Stubble	99.92	97.52	99.55	99.21	99.92	97.29	99.27	99.91	99.95	99.47 ± 0.60
Celery	95.29	61.53	97.54	98.58	99.86	99.39	99.04	98.64	99.11	99.94 ± 0.04
Grapes_untrained	57.00	68.93	73.52	74.23	50.84	71.59	72.61	76.63	72.00	78.77 ± 2.28
Soil_vinyard_develop	90.64	92.83	93.81	97.74	89.03	88.16	99.74	99.58	99.56	99.99 ± 0.01
Corn_sensced_green_weeds	85.87	69.33	77.21	80.54	81.24	69.72	84.51	81.52	86.72	84.10 ± 2.31
Lettuce_romaince_4wk	38.32	59.07	42.37	96.43	89.46	89.29	98.17	97.84	96.59	99.15 ± 0.71
Lettuce_romaince_5wk	87.56	70.59	95.85	99.13	99.17	94.03	99.04	99.56	99.65	99.97 ± 0.04
Lettuce_romaince_6wk	88.66	75.38	99.23	98.61	99.56	99.45	98.97	99.64	99.42	99.12 ± 0.69
Lettuce_romaince_7wk	87.87	89.12	92.98	97.95	98.87	96.58	97.77	98.40	98.19	99.04 ± 0.73
Vinyard_untrained	33.18	47.62	50.37	73.85	59.75	69.30	74.12	73.90	72.65	80.40 ± 4.08
Vinyard_vertical_trellis	81.64	88.90	80.54	88.75	77.69	81.86	90.62	91.34	92.52	87.26 ± 4.4

**Table 6:** Class-specific classification accuracy (%) of different methods for the Indian Pines datasets from the target scene (five TD labeled samples).

Class	SVM	3D-CNN	DFSL	Gai-CFSL	DPGN	RN-FSC	DCFSL	SCFormer-R	SCFormer-S	Ours
Alfalfa	41.30	92.68	97.44	91.60	95.12	96.65	95.61	87.80	93.41	100.00 ± 0.0
Corn-notill	42.86	38.44	38.34	50.46	47.15	45.95	50.44	46.13	50.25	55.02 ± 5.06
Corn-mintill	39.04	44.85	43.35	44.88	27.03	41.25	48.42	45.68	51.84	66.91 ± 4.99
Corn	59.49	36.64	68.45	81.61	56.47	59.06	79.57	64.01	61.77	97.24 ± 2.16
Grass-pasture	59.63	71.13	70.21	70.76	39.75	65.90	73.89	73.87	77.85	84.77 ± 2.57
Grass-Tree	84.79	72.69	76.38	84.25	61.38	69.51	88.26	87.54	90.41	84.50 ± 8.58
Grass-pasture-moved	92.86	100	99.77	97.10	100	99.65	99.57	96.96	99.13	99.13 ± 1.74
Hay-windrowed	90.79	83.93	75.67	91.12	92.39	76.91	88.44	86.15	90	78.73 ± 3.46
Oats	90.00	33.33	99.00	99.26	100	100	100	98.67	98	100.00 ± 0.0
Soybean-notill	34.57	64.84	47.90	62.68	57.91	26.05	61.71	58.42	56.06	72.37 ± 1.47
Soybean-mintill	0.00	58.04	57.80	66.54	41.18	65.36	57.82	64.48	57.87	66.47 ± 4.85
Soybean-clean	15.01	23.64	38.13	42.06	47.96	26.31	40.34	34.46	34.97	45.99 ± 6.59
Wheat	89.76	91.00	98.04	97.11	89.00	99.28	99.25	98.20	95.60	100.00 ± 0.0
Woods	90.91	53..97	83.08	87.10	78.65	75.66	87.26	85.35	86.90	97.17 ± 0.56
Building_Grass-Trees_drives	17.36	56.96	62.86	68.74	46.72	69.90	68.71	66.85	65.62	67.19 ± 7.36
Stone-Stell_Towers	86.02	100	99.94	97.47	98.86	99.88	98.52	99.89	99.77	100.00 ± 0.0

## Data Availability

Data are contained within the article.

## References

[1] Zhang G., Cao W., Wei Y. (2022). Spatial perception correntropy matrix for hyperspectral image classification. Appl. Sci..

[2] Wan S., Gong C., Zhong P., Du B., Zhang L., Yang J. (2019). Multiscale dynamic graph convolutional network for hyperspectral image classification. IEEE Trans. Geosci. Remote Sens..

[3] Jia S., Jiang S., Lin Z., Li N., Xu M., Yu S. (2021). A survey: Deep learning for hyperspectral image classification with few labeled samples. Neurocomputing.

[4] Liang L., Di L., Zhang L., Deng M., Qin Z., Zhao S., Lin H. (2015). Estimation of crop LAI using hyperspectral vegetation indices and a hybrid inversion method. Remote Sens. Environ..

[5] Yang X., Yu Y. (2017). Estimating soil salinity under various moisture conditions: An experimental study. IEEE Trans. Geosci. Remote Sens..

[6] Li S., Dian R., Fang L., Bioucas-Dias J.M. (2018). Fusing hyperspectral and multispectral images via coupled sparse tensor factorization. IEEE Trans. Image Process..

[7] Zhang S., Li J., Wu Z., Plaza A. (2018). Spatial discontinuity-weighted sparse unmixing of hyperspectral images. IEEE Trans. Geosci. Remote Sens..

[8] Tong Q.X., Zhang B., Zhang L.F. (2016). Current progress of hyperspectral remote sensing in China. J. Remote Sens..

[9] Jia S., Hu J., Zhu J., Jia X., Li Q. (2017). Three-dimensional local binary patterns for hyperspectral imagery classification. IEEE Trans. Geosci. Remote Sens..

[10] Li Y., Li Q., Liu Y., Xie W. (2019). A spatial-spectral SIFT for hyperspectral image matching and classification. Pattern Recognit. Lett..

[11] Wang Q., Zhang F., Li X. (2018). Optimal clustering framework for hyperspectral band selection. IEEE Trans. Geosci. Remote Sens..

[12] Wang Q., He X., Li X. (2018). Locality and structure regularized low rank representation for hyperspectral image classification. IEEE Trans. Geosci. Remote Sens..

[13] Ma L., Crawford M.M., Tian J. (2010). Local manifold learning-based k-nearest-neighbor for hyperspectral image classification. IEEE Trans. Geosci. Remote Sens..

[14] Kuo B.C., Huang C.S., Hung C.C., Liu Y.L., Chen I.L. Spatial information based support vector machine for hyperspectral image classification. Proceedings of the 2010 IEEE International Geoscience and Remote Sensing Symposium, IEEE.

[15] Ren Y., Zhang Y., Li L. A spectral-spatial hyperspectral data classification approach using random forest with label constraints. Proceedings of the 2014 IEEE Workshop on Electronics, Computer and Applications.

[16] Wang J.X., Chen S.B., Ding C.H., Tang J., Luo B. (2021). RanPaste: Paste consistency and pseudo label for semi-supervised remote sensing image semantic segmentation. IEEE Trans. Geosci. Remote Sens..

[17] Chen Y., Zhao X., Jia X. (2015). Spectral–spatial classification of hyperspectral data based on deep belief network. IEEE J. Sel. Top. Appl. Earth Obs. Remote Sens..

[18] Li Y., Zhang H., Shen Q. (2017). Spectral–spatial classification of hyperspectral imagery with 3D convolutional neural network. Remote Sens..

[19] Thompson S., Teixeira-Dias F., Paulino M., Hamilton A. (2022). Ballistic response of armour plates using generative adversarial networks. Def. Technol..

[20] Zhong Z., Li J., Luo Z., Chapman M. (2017). Spectral–spatial residual network for hyperspectral image classification: A 3-D deep learning framework. IEEE Trans. Geosci. Remote Sens..

[21] Huang L., Chen Y. (2020). Dual-path siamese CNN for hyperspectral image classification with limited training samples. IEEE Geosci. Remote Sens. Lett..

[22] Shendryk Y., Rist Y., Ticehurst C., Thorburn P. (2019). Deep learning for multi-modal classification of cloud, shadow and land cover scenes in PlanetScope and Sentinel-2 imagery. ISPRS J. Photogramm. Remote Sens..

[23] Zheng X., Jia J., Chen J., Guo S., Sun L., Zhou C., Wang Y. (2022). Hyperspectral image classification with imbalanced data based on semi-supervised learning. Appl. Sci..

[24] Yang Y., Tang X., Zhang X., Ma J., Liu F., Jia X., Jiao L. (2022). Semi-supervised multiscale dynamic graph convolution network for hyperspectral image classification. IEEE Trans. Neural Netw. Learn. Syst..

[25] Datta D., Mallick P.K., Bhoi A.K., Ijaz M.F., Shafi J., Choi J. (2022). Hyperspectral image classification: Potentials, challenges, and future directions. Comput. Intell. Neurosci..

[26] Wang X., Tan K., Du P., Pan C., Ding J. (2022). A unified multiscale learning framework for hyperspectral image classification. IEEE Trans. Geosci. Remote Sens..

[27] Huang G., Liu Z., Van Der Maaten L., Weinberger K.Q. Densely connected convolutional networks. Proceedings of the IEEE Conference on Computer Vision and Pattern Recognition.

[28] Wang X., Fan Y. (2022). Multiscale densely connected attention network for hyperspectral image classification. IEEE J. Sel. Top. Appl. Earth Obs. Remote Sens..

[29] He K., Zhang X., Ren S., Sun J. Deep residual learning for image recognition. Proceedings of the IEEE Conference on Computer Vision and Pattern Recognition.

[30] Dhillon G.S., Chaudhari P., Ravichandran A., Soatto S. (2019). A baseline for few-shot image classification. arXiv.

[31] Mathivanan P., Maran P. (2024). Color image encryption based on novel kolam scrambling and modified 2D logistic cascade map (2D LCM). J. Supercomput..

[32] Devabathini N.J., Mathivanan P. Sign Language Recognition Through Video Frame Feature Extraction using Transfer Learning and Neural Networks. Proceedings of the 2023 International Conference on Next Generation Electronics (NEleX).

[33] Koch G., Zemel R., Salakhutdinov R. Siamese neural networks for one-shot image recognition. Proceedings of the ICML Deep Learning Workshop.

[34] Vinyals O., Blundell C., Lillicrap T., Wierstra D. Matching networks for one shot learning. Proceedings of the 30th International Conference on Neural Information Processing Systems.

[35] Ren M., Triantafillou E., Ravi S., Snell J., Swersky K., Tenenbaum J.B., Larochelle H., Zemel R.S. (2018). Meta-learning for semi-supervised few-shot classification. arXiv.

[36] Munkhdalai T., Yu H. Meta networks. Proceedings of the International Conference on Machine Learning.

[37] Sun Q., Liu Y., Chua T.S., Schiele B. Meta-transfer learning for few-shot learning. Proceedings of the IEEE/CVF Conference on Computer Vision and Pattern Recognition.

[38] Yu M., Guo X., Yi J., Chang S., Potdar S., Cheng Y., Tesauro G., Wang H., Zhou B. (2018). Diverse few-shot text classification with multiple metrics. arXiv.

[39] Liu Y., Sun Q., Liu A.A., Su Y., Schiele B., Chua T.S. (2019). LCC: Learning to customize and combine neural networks for few-shot learning. arXiv.

[40] Liu B., Yu X., Yu A., Zhang P., Wan G., Wang R. (2018). Deep few-shot learning for hyperspectral image classification. IEEE Trans. Geosci. Remote Sens..

[41] Li J., Zhang Z., Song R., Li Y., Du Q. (2024). SCFormer: Spectral Coordinate Transformer for Cross-Domain Few-Shot Hyperspectral Image Classification. IEEE Trans. Image Process..

[42] Zhang S., Chen Z., Wang D., Wang Z.J. (2022). Cross-Domain Few-Shot Contrastive Learning for Hyperspectral Images Classification. IEEE Geosci. Remote Sens. Lett..

[43] Misra D. (2019). Mish: A self regularized non-monotonic activation function. arXiv.

[44] Indian Pines Dataset. https://www.ehu.eus/ccwintco/index.php/Hyperspectral_Remote_Sensing_Scenes.

[45] Yokoya N., Iwasaki A. (2016). Airborne Hyperspectral Data over Chikusei.

[46] Lee H., Kwon H. (2017). Going deeper with contextual CNN for hyperspectral image classification. IEEE Trans. Image Process..

[47] Liu B., Yu A., Gao K., Wang Y., Yu X., Zhang P. (2022). Multiscale nested U-Net for small sample classification of hyperspectral images. J. Appl. Remote Sens..

[48] Peng Y., Liu Y., Tu B., Zhang Y. (2023). Convolutional Transformer-Based Few-Shot Learning for Cross-Domain Hyperspectral Image Classification. IEEE J. Sel. Top. Appl. Earth Obs. Remote Sens..

[49] Gao K., Liu B., Yu X., Qin J., Zhang P., Tan X. (2020). Deep relation network for hyperspectral image few-shot classification. Remote Sens..

[50] Zhang Y., Li W., Zhang M., Wang S., Tao R., Du Q. (2022). Graph information aggregation cross-domain few-shot learning for hyperspectral image classification. IEEE Trans. Neural Netw. Learn. Syst..

[51] Yang L., Li L., Zhang Z., Zhou X., Zhou E., Liu Y. Dpgn: Distribution propagation graph network for few-shot learning. Proceedings of the IEEE/CVF Conference on Computer Vision and Pattern Recognition.

[52] Li Z., Liu M., Chen Y., Xu Y., Li W., Du Q. (2022). Deep Cross-Domain Few-Shot Learning for Hyperspectral Image Classification. IEEE Trans. Geosci. Remote Sens..

